# URBANIZATION, FINANCIAL SOURCE, AND PLACE OF DEATH AMONG CHINESE LONGEVITY OLDER ADULTS

**DOI:** 10.1093/geroni/igac059.2509

**Published:** 2022-12-20

**Authors:** Rui Zhou, Shuangshuang Wang, Nengliang Yao

**Affiliations:** Shandong University, Jinan, Shandong, China (People's Republic); Shandong University, Jinan, Shandong, China (People's Republic); Shandong University, Jinan, Shandong, China (People's Republic)

## Abstract

The research on place of death among Chinese oldest adults is scare. This study explored the associations of urbanization and financial source on the places of death among Chinese longevity older adults. The sample consists of 21,920 decedents (female=60%, died at home=89%, mean age at death=95) from 2000-2018 Chinese Longitudinal Healthy Longevity Survey (CLHLS), conducted by Peking University. Places of death were divided into two categories: home and other sites (including hospitals, institutions and others). Urbanization was measured by residential areas: rural or city. Financial source consisted of family financial support and retirement wage. Chi-square test showed that older adults living in rural areas and getting family finance support were more likely to die at home. Results from binary regression models show that controlling for covariates, decedents living in cities were 6.2 times more likely to die in other places than at home and those received retirement wage were 3.1 times respectively. Notably, older adults who lived in cities and also had retirement wage were 17.7 times more likely to die in other places comparing to those living in rural areas with only family finance support. Findings suggest that more older adults will choose to die at hospitals or institutions as the process of urbanization and the development of social pension system in China, which will put enormous pressure to the hospital-centered healthcare system. Improving the quality of healthcare in grassroots areas might be a feasible attempt to relive the pressure of hospital-centered healthcare system in cities.

